# Tumor microenvironment: barrier or opportunity towards effective cancer therapy

**DOI:** 10.1186/s12929-022-00866-3

**Published:** 2022-10-17

**Authors:** Aadhya Tiwari, Rakesh Trivedi, Shiaw-Yih Lin

**Affiliations:** 1 Department of Systems Biology, The University of Texas MD Anderson Cancer Center, Houston, TX USA; 2 Department of Translational Molecular Pathology, The University of Texas MD Anderson Cancer Center, Houston, TX USA

**Keywords:** Tumor microenvironment, Cancer immunotherapy, 3D models, Checkpoint inhibitors, Specialized microenvironment, TME profiling, CAR-T/NK therapy

## Abstract

Tumor microenvironment (TME) is a specialized ecosystem of host components, designed by tumor cells for successful development and metastasis of tumor. With the advent of 3D culture and advanced bioinformatic methodologies, it is now possible to study TME’s individual components and their interplay at higher resolution. Deeper understanding of the immune cell’s diversity, stromal constituents, repertoire profiling, neoantigen prediction of TMEs has provided the opportunity to explore the spatial and temporal regulation of immune therapeutic interventions. The variation of TME composition among patients plays an important role in determining responders and non-responders towards cancer immunotherapy. Therefore, there could be a possibility of reprogramming of TME components to overcome the widely prevailing issue of immunotherapeutic resistance. The focus of the present review is to understand the complexity of TME and comprehending future perspective of its components as potential therapeutic targets. The later part of the review describes the sophisticated 3D models emerging as valuable means to study TME components and an extensive account of advanced bioinformatic tools to profile TME components and predict neoantigens. Overall, this review provides a comprehensive account of the current knowledge available to target TME.

## Background

Significant developments made in the field of cancer therapy over the last decade have led to the improvement in the life expectancy of cancer patients [1]. However, these approaches are not equally effective across different tumor types and patients. Even the response to a specific line of therapy varies broadly depending on the type of tumor, i.e., benign, locally advanced or metastatic [2]. These therapeutic challenges have been exceedingly attributed to the ability of cancer cells to hijack the host machinery to create a niche, i.e., tumor microenvironment for themselves, and progressively modulate it from anti-tumoral to pro-tumoral responses [3]. Furthermore, a lack of unanimity in defining TME in tumor proximal and distal locations, and unavailability of model systems that accurately mimic the interaction of cancer cells with its microenvironment are the two prime reasons for our limited understanding of TME and exploring its therapeutic potentials [4, 5].

Tumor microenvironment is an ecosystem created by the cancer cells and comprised of components contributed by both tumor and host. Different factors in the TME unanimously ensure the development, progression and expansion of tumor through an uninterrupted supply of nutrients and oxygen, hampering the immune surveillance reach, and efficient drugs carting [5]. A dynamic interaction between cancer cells with these cellular and acellular components of TME is essential for generating heterogeneity, clonal evolution and enhancing multi drug resistance in tumor cells [6]. Broadly, six distinct specialized microenvironments within TME have been identified namely the hypoxic niche, acidic niche, innervated niche, metabolism microenvironment, immune microenvironment, and mechanical microenvironment [4].

Presently, most of the therapeutic efforts involving the TME either focusses on targeting TME components or the development of experimental model systems that accurately mimics complexities of TME. Moreover, TME-targeting strategies can be achieved by stimulating the intrinsic host immune system, which encompass either activation of anti-tumor immune cells or inhibiting pro-tumoral immune cells in the TME. Recent studies have shown that these specialized TME microenvironments and niches have potential to act as target of cancer therapy through reprogramming [7–22].

Various modes of cancer treatment have been applied in clinic, which include surgery, chemotherapy, hormone therapy, radiation therapy, immunotherapy, targeted therapy, hyperthermia, photodynamic therapy and stem cell transplant [23]. More recently, immunotherapy has emerged as the preferred regimen because of its ability to induce durable responses, and low degrees of side-effects as compared to other types of treatment methods [24]. Cancer immunotherapy (CIT) strategies include adoptive T-cell transfer, immune checkpoint inhibitors, monoclonal antibodies, non-specific immune stimulation, oncolytic virus immunotherapy and vaccinations [25]. The major challenges posed by CIT approaches include lack of therapeutic responses and acquired resistance [26]. Therefore, lack of understanding of real-time TME dynamics and identification of cancer specific neoantigen is the major challenge in developing effective immunotherapy. Advanced 3D models provide the opportunity to reconstruct and comprehend the heterogeneous TME and its dynamic interactions more precisely by using human cells, and tightly regulated cellular compositions, physiological conditions and physical parameters [27–31]. Furthermore, bioinformatic tools are emerging as prospective means to profile and predict neoantigen. In this review, we described different components of TME as barrier and opportunity to target cancer, the 3D models available to understand TME, and the bioinformatics advancements towards profiling of tumor microenvironments for identification of novel immunotherapeutic targets.

## Salient features of TME

**Fig. 1:**
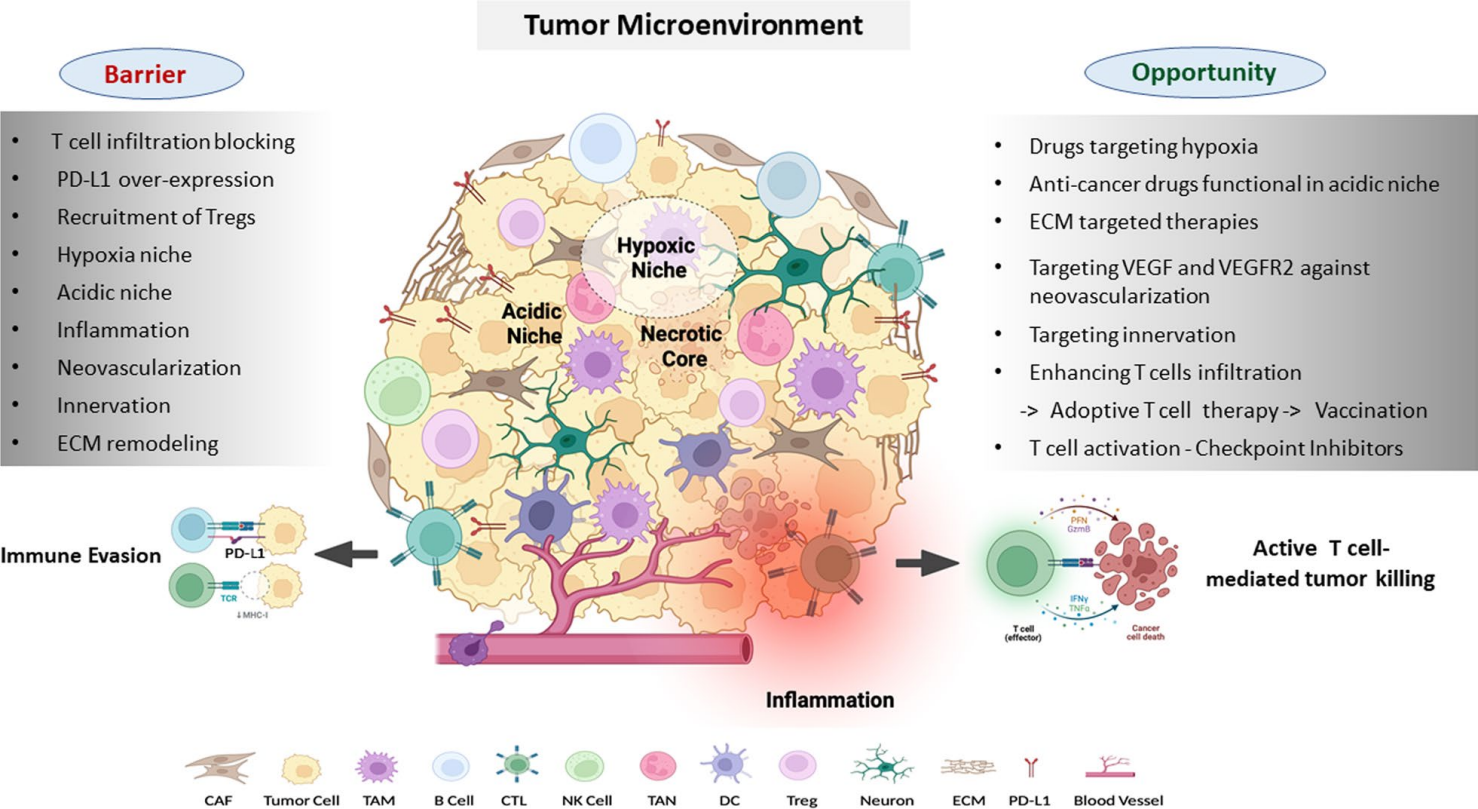
Tumor microenvironment (TME): TME is a complex ecosystem of cellular. niche, acidic niche, inflammation etc. Extracellular matrix (ECM), the major non-cellular and acellular components, and several specialized microenvironments such as hypoxic component, provides architectural support, and act as a store house for factors such as chemokines, cytokines, growth factors etc., required for continuous tumor transformation process. Cellular components consist of non-immune and immune cell populations. Non- immune cell types include tumor cell, cancer associated fibroblast (CAF), neuron, and endothelial cell (blood vessel) that helps in tumor invasion, progression, and metastasis. Immune cells within TME comprise of tumor-associated macrophages (TAMs), tumor- associated neutrophils (TANs), dendritic cell (DCs), regulatory T cell (Treg), B cell, Natural killer (NK) cell, and cytotoxic T lymphocytes (CTLs). In immuno-competent conditions, CTL identifies and bring about tumor cell killing by releasing cytotoxic molecules such as granzyme-B, interferon-γ (IFN-γ), perforins etc. The figure is prepared by using BioRender software and publication license is obtained

The main elements which define the variability in the TME includes but are not limited to genomic instability, tumor type, tumor location, presence of mutations such as KRAS, EGFR, PTEN etc., presence of lymph nodes, adipose tissue in the vicinity, and therapeutic interventions [32]. However, there are certain characteristics which are constant and encompass the hallmarks of the TME. These hallmarks include the presence of stromal cells, endothelial cells, components of both innate and adaptive immune cells and extracellular matrix [33]. Altogether, the intricate interactions among these TME elements led to the development of localized and specified microenvironments within TME which in turn defines the resilience and immunogenicity of the tumor as summarized in Fig. 1.

### Specialized microenvironments of the TME

#### Hypoxic niche

**Fig. 2:**
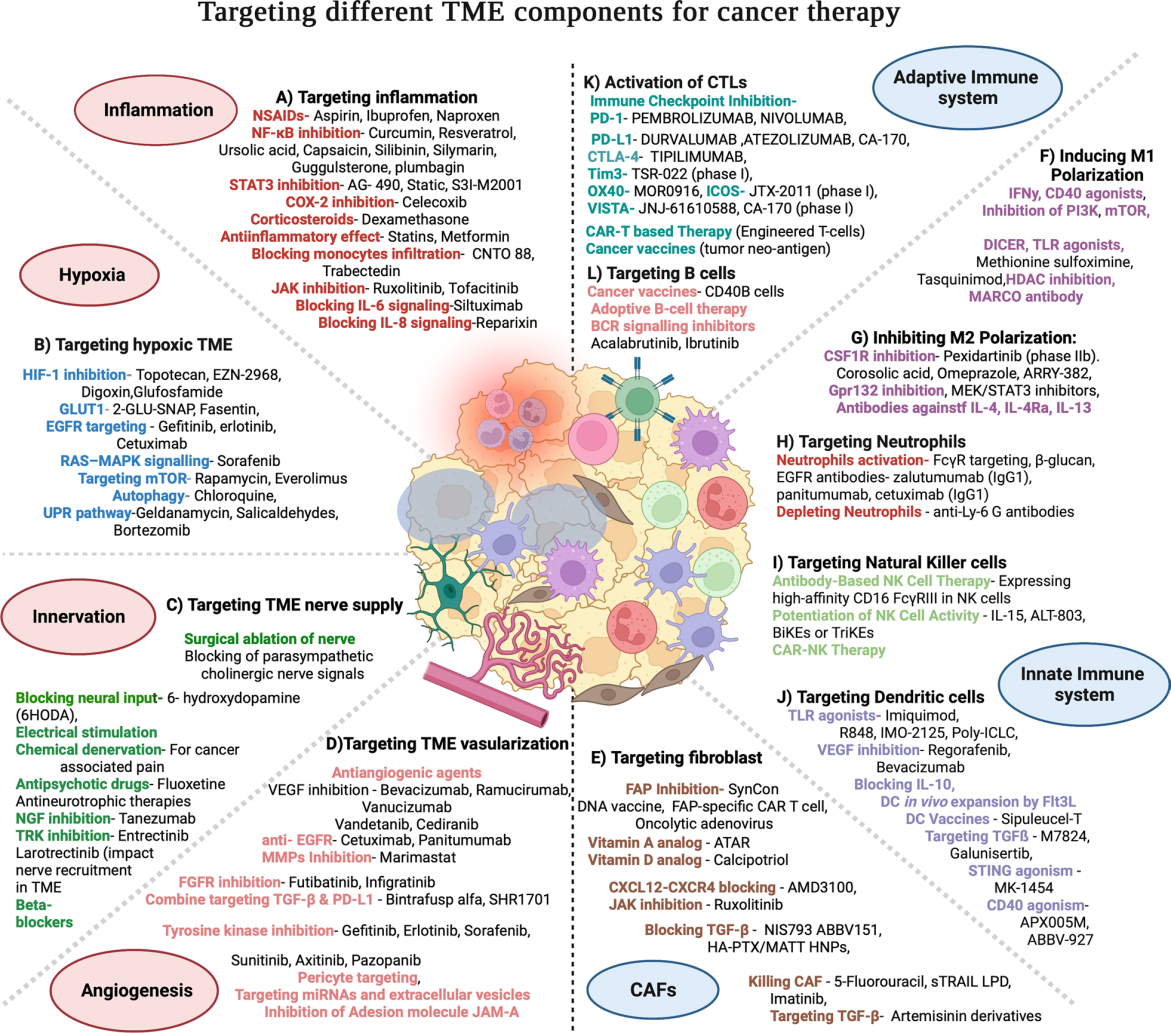
Targeting different TME components for cancer therapy: Current strategies available for targeting major TME components for effective cancer therapy are shown. **A** Targeting inflammation, **B** targeting hypoxic TME, **C** targeting TME nerve supply, **D** targeting TME vascularization and cellular components like **E** targeting cancer associated fibroblasts (CAFs), targeting innate immune components by **F** Inducing M1Polarization, **G** Inhibiting M2 Polarization, **H** targeting Neutrophils, **I** targeting Natural Killer cells, **J** targeting Dendritic cells, and targeting adaptive immune components by **K** Activation of CTLs and **L** Targeting B cells are promising targets. Various drugs/inhibitors/antibodies targeting these components are in preclinical studies, under clinical trial or FDA approved for cancer treatment. The figure is prepared by using BioRender software and publication license is obtained

Uncontrolled proliferation of cancer cells and limited vascularization from host cells give rise to oxygen crisis in different areas of a tumor [34]. These oxygen-restricted areas of the TME are known as the hypoxic niche as depicted in Fig. 1. The adaptation of cancer cells to a hypoxic environment is largely mediated by hypoxia inducible factor-1 (HIF-1) [35]. HIF-1 initiates the angiogenic process through activation of multiple factors including the most prominent angiogenic ligand, vascular endothelial growth factors (VEGF), and its receptors including VEGFR2 [36]. HIF-1 regulates expression of cancer stem cell markers like KLF4, MYC, OCT4, SOX2, and NANOG, and thus help cancer cells to survive through hypoxic crisis [37–39]. In addition to pluripotent factors, HIF-1 also regulates other epigenetic modifiers involved in regulating stemness, including BMI1 and SIRT1 [40]. Hypoxia signaling can promote EMT mainly through HIF-1-mediated transactivation of EMT inducing factors (EIFs) such as TWIST, SNAIL, and ZEB1 [41]. In addition, HIF-1 can also activate transforming growth factor beta (TGF-β), WNT, and NOTCH signaling and inhibit the Hippo signaling pathway to promote survival of cancer stem cells [42–45]. Several compounds capable of inhibiting hypoxia inducible factor-1 (HIF-1) or its targets have shown competence in inhibiting tumor progression and are in clinical trials Fig. 2B. For instance, topotecan, a topoisomerase 1 inhibitor, has been approved by the US Food and Drug administration (FDA) to be used as a second line of treatment for small cell lung and ovarian cancers [46]. Another drug targeting the hypoxic niche of TME, metformin, is presently in a clinical trial for head and neck squamous cell carcinoma (NCT03510390) [47]. Moreover, several hypoxia-reactive prodrugs, which become activated in the TME’s hypoxic niche have been developed [48]. TME also uses hypoxic response to rewire the metabolic mechanisms and transcriptomic profiles, which can be used as a therapeutic target in combinatorial therapy [49, 50]. Wigerup et al. has described various strategies to target hypoxia, and the drugs under exploration in a comprehensive review [51].

#### Acidic microenvironment

Tumor cells prefer glycolysis as the major mode of glucose metabolism, even in the presence of oxygen [52, 53]. Hypoxia and reduced vascularization further triggers glycolysis and suppress oxidative phosphorylation in tumor tissue. The elevated level of glycolysis causes increased lactate accumulation in the TME, which results in the extracellular low pH [54]. Acidic microenvironment at its initial stage of formation acts as a hostile niche, and triggers apoptosis in cancer cells. However, persistent TME acidification prompts cancer-cell adaptation, and results in aggressive tumors and hence act as barrier for effective therapy [55]. Furthermore, low pH facilitates activation of pro-tumorigenic macrophages, neutrophils, dendritic cells (DCs), and inhibition of tumor-infiltrating lymphocytes (TILs) cytotoxic activity, hence impairing immunosurveillance [4]. Targeting dysregulated pH zones of TME is therefore a potential opportunity for effective therapeutic intervention (Fig. 1). Several small-molecule inhibitors to target the acidic niche of TME are under exploration. In addition, pH-responsive drug release systems have also been recently developed to deliver cytotoxic drugs to the acidic TME. Recently, several small molecular inhibitors have been developed to target acidic tumor microenvironment, and Zhong et al. provides a detailed description of such class of inhibitors [56].

#### Inflammatory microenvironment

Inflammation plays critical role in initiation, progression and metastasis of cancer [57, 58]. The chemo-attractants produced by cancer cells stimulates the infiltration of immune cells like neutrophils, macrophages, dendritic cells, eosinophils and mast cells in TME. These inflammatory cells secrete pro-inflammatory cytokines like IL-1, IL-6, IL-15, IL-17, IL-23, tumor necrosis factor-α (TNF-α), and other molecules like IFN-γ, reactive oxygen species (ROS), serine and cysteine proteases, matrix metalloproteinases and membrane-perforating agents which are cytotoxic to tumor cells. Inflammation also potentiates the proliferation of residential myeloid cells and enhance the secretion of inflammatory factors such as histamines, cytokines etc., within TME. Additionally, it activates adaptive immune cells, and results in recruitment of anti-tumorigenic cytotoxic T lymphocytes (CTLs) [59]. Acute inflammation therefore creates a hostile condition for the tumor growth and progression [60–62]. However, persistent inflammation, hypoxic condition and a nutrient-restricted microenvironment within tumor results in the formation of an immunosuppressive microenvironment through accumulation of large number of immunosuppressive cells like M2 macrophages, MDSCs, Treg cells, Breg cells etc., These cells secrete pro-tumor cytokines such as IL-6, IL-1β, IL-17, IL-11, and growth factors like TNF-α which promotes tumor growth, proliferation,metastasis [63, 64] and therapy resistance which act as a barrier for effective therapeutic intervention (Fig. 1). Furthermore, multiple signaling pathways, such as NF-kB, JAK-STAT, TLR pathways, cGAS/STING, and MAPK pathways are known to play important role in regulating pro-tumor inflammation [59]. Targeting inflammatory microenvironment is therefore a potential opportunity, and at present, many anti-inflammatory drugs are undergoing clinical trials (Fig. 2A). One such drug, Statins have shown promising anti-tumor effect in different cancer types including CRC, breast cancer etc. [65, 66]. Aspirin, a non-steroid anti-inflammatory drug (NSAID) have shown beneficial effect in many kinds of cancer [67, 68]. Sulindac, another NSAIDs and selective COX-2 inhibitors are given to patients who are at high risk of getting colorectal cancer [68]. Targeting IL-6 is also emerging as an attractive strategy for cancer prevention and Tocilizumab, an IL-6R-specific antibody is under clinical trials. Chimeric monoclonal antibody siltuximab that binds IL6 is currently investigated for the treatment of several tumor types including prostate cancer and metastatic renal cell cancer. Several natural anti-inflammatory agents like curcumin, resveratrol, ursolic acid, capsaicin, silibinin, silymarin, guggulsterone, and plumbagin have also been enormously explored in cancer prevention. The recent review by Zhao et al. has comprehensively described the tumor-associated inflammation, cytokines/chemokines involved, and the targeting drugs under clinical trials [59].

#### Innervated microenvironment

Neuronal involvement in promoting tumor progression and metastasis is another level of complexity within TME. Intra-tumoral nerves are either newly formed or recruited fibers that infiltrate the TME from nearby tissues [19, 69]. Moreover, neuronal progenitor cells migrate from the brain to home in the developing tumor [70]. These progenitor cells facilitate tumoral neurogenesis through the formation and recruitment of functional neurons within the tumor [20, 71]. Additionally, TME also secretes chemokines that stimulates the nearby nerves to sprout and grow in the tumor [72, 73]. Collectively, this phenomenon has been termed as tumor innervation, and is found to be associated with an aggressive tumor phenotype, cancer-related pain, and poor prognosis in clinical studies [19, 74, 75]. Innervation within TME relies on the release of neurotransmitters or neuropeptides, such as dopamine, catecholamine, and acetylcholine. Crosstalk between the nerves and other TME components such as ECM, immune cells, stromal cells, endothelial cells and tumor cells has also been coupled with the poor prognosis and acquired resistance to current targeted therapies [76]. Therefore, assessment of tumor innervation might serve as a potential predictive marker of disease severity. Some studies have shown that surgically severing of nerves entering the tumor can inhibit tumor growth and metastasis [19, 77, 78]. Anti-neurotrophic therapies have also shown the promising outcomes in targeting densely innervated therapy-resistant tumors (Fig. 2). However, targeting TME innervation is relatively new field, and further studies are required to clearly understand the role of nerves in tumor progression. In a recent review, Li et al. describes the present status, future perspectives and the associated challenges associated with targeting of tumor innervation [79].

#### Tumor vascularization

Tumor-associated dysfunctional and leaky blood vessels regulate tumor perfusion and maintains the immunosuppressive microenvironment necessary for tumor survival and progression. The abnormal morphology and behavior of these tumor blood vessels is because of the disorganized network of the tortuous endothelial cells and lack of the normal hierarchical artery-arteriole-capillary arrangement [80, 81]. However, irrespective of its tissue of origin, several critical functions such as regulation of transport of oxygen, nutrients and other solutes from bloodstream to tissues, maintaining blood flow by providing non-thrombogenic surface, and controlling the infiltration of leukocytes between tissues has been attributed to these endothelial cell populations [82]. Hence, under malignancies, inhibiting the formation of such dysfunctional and leaky blood vessels, interrupting the supply of oxygen and nutrients, and increasing the leukocytes infiltration could be a promising therapeutic intervention (Fig. 1). Therefore, tumor-associated endothelial cells can act as an attractive target for therapeutic purposes [83]. Several anti-angiogenic compounds have been developed and tested in clinical trials (Fig. 2D); the results are promising with an increase in the overall survival. Bevacizumab (Avastin), the first anti-angiogenic antibody approved by the FDA is already in use [84]. Furthermore, sunitinib (Sutent), a multi-tyrosine kinase inhibitor of VEGF, platelet-derived growth factor receptor (PDGFR), and receptor tyrosine kinase—oncogene c-KIT (KIT) is a potent anti-angiogenic drug approved for the treatment of various tumors [85]. Recent review by Zheng et al. has comprehensively described various approaches employed in targeting tumor vascularization [86].

### Major components of TME and targeting strategies

#### Stromal cells

Cancer cells and other TME cells secretes growth factors like transforming growth factor beta (TGF-β), fibroblast growth factor 2 (FGF2), platelet-derived growth factor (PDGF), and epidermal growth factor (EGF) which are key regulators of fibroblast recruitment and activation [87, 88]. Cancer associated fibroblasts (CAFs) provide the physical support to the cancer cells by secreting ECM (Fig. 1). CAFs also secrete MMPs, which are ECM-degrading proteases; hence regulates ECM turnover and plays crucial role in tumor invasion [89]. Additionally, CAFs also play a critical role in the angiogenesis, progression and metastasis of tumors by producing many growth factors and proinflammatory cytokines like vascular endothelial growth factor (VEGF), IL-6, CXC-chemokine ligand (CXCL12), TGFβ, NF-κB, TNF-α, IFN-γ, SDF-1α, EGF, galectin-1 [90–95]. CAFs therefore are the key determinant in the tumor development, and are emerging as a potential target for cancer therapies [96] as described in Fig. 2. One of the most successful approaches is targeting fibroblast activating protein (FAP) expressed by stromal cells. FAB is known to be involved in the proliferation of stromal cells as well as ECM secretion [97, 98]. Anti-FAB antibody conjugated with cytotoxic drug like maytansine (DM1) has shown promising results in xenograft mice models studies [99]. Besides, all-trans retinoic acid (ATRA) has also been observed to inhibit FAP, TGFβR and αSMA expression in CAFs, and therefore, currently being used for the treatment of multiple cancer types in conjunction with other drugs [100, 101]. Furthermore, drugs-like paricalcitol, a vitamin D receptor agonist, has been used to reprogram pro-tumor CAFs to a quiescent-like state [102]. In a comprehensive review, Chen et al. describes various strategies available to target CAFs, and different drugs under evaluation presently [103].

#### Extracellular matrix (ECM)

Extracellular matrix (ECM) is the major non-cellular component of TME and mainly comprise of collagen. In addition to providing structural framework, ECM also acts as a store house of several chemokines, cytokines and growth factors which plays important role in creating immune-suppressive tumor microenvironment [104]. Approximately 60% of the total mass of the solid tumors are comprised of ECM deposits that provides structural stiffness to them. Such ECM rigidity enables the cancer cells to proliferate aggressively and undergo directed cell migration [105, 106]. High matrix stiffness has been observed in aggressive tumors such as triple negative breast cancer and associated with poor prognosis [107] (Fig. 1). TGF-β, which regulates collagen synthesis, has been shown to inhibit tumor growth in several in vitro studies [108–110]. Fresolimumab, a monoclonal antibody that targets TGF-β mediated collagen synthesis, underwent several clinical trials (clinicaltrials.gov identifier: NCT01401062 and NCT02581787) [111–113]. Similarly, ronespartat (SST0001), showed strong anti-tumor effect in multiple myeloma in a Phase 1 clinical trial (Clinical Trial NCT01764880) [114]. Since the high expression of MMPs in the TME regulates ECM homeostasis and facilitates tumor invasion and metastasis, MMPs inhibitors have also been evaluated as potential therapeutic target and have shown promising results [115]. Drugs targeting MMPs like incyclinide, have been through several clinical trials for AIDS-related sarcomas (Clinical trials NCT00004147, NCT00003721, NCT00001683, and NCT00020683) [116]. Other MMPs-targeting agents include JNJ0966, which is highly selective towards MMP-9, and the antibody Fab 3369, which targets MMP-14 [117, 118]. In a recent review, Huang et al. has described various ECM targeting strategies in detail [119].

#### Major immune components—innate and adaptive

Immune cells are essential components of TME. Depending on the stage and complexity of the TME, immune cells can either be anti-tumorigenic or promote tumor growth. Chronic inflammation at the site of tumor growth activates the residential and circulatory immune cells to accumulate in the vicinity of tumor. Furthermore, components of tumor microenvironment especially stromal cells secrete cytokines and chemokines which attract infiltration of immune cells from both innate and adaptive immune system. Various review articles discussing diverse immune components of TME in detail are widely available in literature, hence, the description of immune components of TME is kept concise in the current review [33, 120–126].

##### Macrophages

Macrophages are specialized cells of the innate immune system that are derived from monocytes. Several soluble factors secreted by the cancer or stromal cells are responsible for recruitment of macrophages within TME. These soluble molecules include IL-3, colony stimulating factor 1 (CSF-1), and CCL2 [61]. CSF-1 induce monocyte transformation into highly plastic non-polarized (M0) macrophages. M0 macrophages can be stimulated by interferon-γ (IFN-γ) into activated anti-tumor M1 macrophages which exerts cytotoxic effect on tumor by secreting cytokines like IL-2 and TNFα. Conversely IL-4, IL-10 and IL-13 secreted by tumor or stromal cells can stimulate conversion of M0 to pro-tumor M2 macrophages. These tumor-associated macrophages (TAMs) secrete anti-tumor cytokines IL-6, IL-8 and IL-10 and matrix metalloproteinases (MMPs) which regulates neo-vasculogenesis in TME [127]. The cytokines and chemokines secreted by macrophages and tumor cells within TME further impede CD8 + T-cell infiltration, thus creating an immunosuppressive microenvironment which support tumor growth and metastasis [61]. High infiltration of TAM within TME has been shown to be associated with poor prognosis in many tumor types [128–131]. Targeting macrophages therefore is a promising strategy verified in many clinical trials; however complete removal of macrophages has shown severe liver toxicity. Various indirect approaches to target TAMs are presently under evaluation in several studies Fig. 2F, G. For example, targeting colony stimulating factor by CSF1R inhibitors alone or in combination with other agents have shown promising anti-tumor efficacy in various tumor types. Several studies targeting pro-inflammatory CCR2/CCL2 axis have shown initial promising outcomes. PF-04136309 (CCR2 inhibitor) is in a phase 1 clinical trial and has been tested in combination with FOLFIRINOX chemotherapy in PDAC patients [132]. Similarly, changing the polarization of pro-inflammatory M2 to anti-inflammatory M1 state within TME can enhance the anti-tumor effect of TAMs, and such strategy is under evaluation in several clinical trials. Recent developments regarding therapeutic targeting of macrophages within TME have been extensively described in the review by Pathriaet et al. [133].

##### Neutrophils

Tumor-associated inflammation drives accumulation of neutrophils within TME. The chemokine/cytokine composition of the TME determines the anti-tumor (N1) or pro-tumor (N2) phenotype of tumor-associated neutrophils (TANs). Anti-tumor N1 TANs possess high levels of TNFα, CCL3, ICAM-1, and are also involved in the production of ROS which is cytotoxic to tumor cells. In the later stages of tumor development, there is high infiltration of N2 neutrophils that support tumor growth and progression [134, 135]. IL-8 secreted by tumor cells in the TME stimulates neutrophils to release arginase enzyme which is responsible for degradation of arginine. Arginine is essential for the activation and proliferation of T-cell. Hence, TANs play important role in suppression of T-cell immune response. Additionally, TANs also suppress NK cells activation by regulating the secretion of IL-18. Tumor-associated neutrophils (TANs) therefore contribute substantially to tumor progression, invasion, and angiogenesis [136, 137]. A comprehensive account of targeting TAN in cancer immunotherapy has been recently described by Rahmy et al. [138].

##### Dendritic cells

Tumor infiltrating dendritic cells (DCs) are the chief antigen-presenting cells (APCs) which scan and phagocytose the tumor associated antigens, process the antigen peptide to present with MHC class II molecule and prime CD8 + T cells. The pro-inflammatory cytokines and other soluble factors present in TME such as, IL-15, IL-2, IL-21, IFN-α, and GM-CSF further enhance the anti-tumor characteristic of DC.

Tumor cells and other TME components like stromal cell, endothelial cells, tumor infiltrating immune cells also secrete cytokines, chemokines, prostaglandins and growth factors which can modulate the DCs to behave in a pro-tumor fashion. These soluble factors like IL-6, IL-10, IDO, M-CSF, TGF-β1, PGE2, VEGF present in TME reprogram DCs to possess inefficient antigen-presenting capabilities, and an immunosuppressive regulatory phenotype that supports tumor progression [139–143]. The balance between the levels of different cytokines and growth factors in TME determines the pro-tumor or anti-tumor nature of DC. Targeting these cytokines therefore can be promising strategy to potentiate the immunotherapies like immune checkpoint blockade and CAR-T therapy. Personalized vaccines comprise of patient derived DCs which are engineered and amplified and injected back to host circulation have shown promising tumor suppressing effect [144] Fig. 2J. Similarly, delivery of ligand for toll-like receptor 3 (TLR3) or STING agonist to activate DCs at the site of tumor are showing promising result in enhancing DC based immune response [145]. Furthermore, combination therapy of DC vaccines with anti-inflammatory drug like aspirin have shown promising outcome in pre-clinical models [146]. Wculek et al. has described the role of dendritic cells in TME and DC-targeting strategies in a comprehensive review [147].

##### Natural killer cells

Natural killer (NK) cells are key components of the innate immune system and are highly efficient in identifying and killing undifferentiated or poorly differentiated tumor cells in the tumor or in circulation [148, 149]. The major mode of action of NK cells is by releasing perforins and granzyme B to induce necrotic or apoptotic cell death. NK cells secrete a wide variety of anti-tumor cytokines such as IL-10, IL-5, IL-13, GM-CSF, IFN-γ, TNF-α [150, 151]. IFN-γ is one of the major anti-tumor cytokines secreted by NK cells. The balance between the cytokines secreted by tumor infiltrating immune cells, tumor cells or stromal cells in the tumor microenvironment determines the pro-tumor or anti-tumor characteristic of NK cells [152]. Immunosuppressive factors secreted by tumor cells include TGF-β, VEGF, indoleamine 2,3-dioxygenase (IDO), prostaglandin E2 (PGE2), and adenosine which inhibit antitumor immune functions. Tregs, MDSC, and M2-macrophages secrete immunosuppressive cytokines such as IL-10 and TGF-β which inhibits NK cells activation and function. Therefore, enhancing the NK cells activity within the TME is a promising direction in cancer therapy [153, 154] Fig. 2I. Wu et al. has provided a detailed description of NK cell-based targeting strategies for cancer therapy [153].

##### T cells

T-cells forms the major line of anti-tumor adaptive immune response in TME. Tumor-infiltrating lymphocytes (TILs) present in TME include CD4 + helper cells, immunosuppressive CD4 + FOXP3 + regulatory T-cells (Tregs) and CD8 + cytotoxic T-cells (CTLs). Higher CD8 + T cells infiltration is mostly associated with better prognosis and therapy response. Naive CD8 + T cells gets activated and differentiate into cytotoxic effector T cells on encountering tumor-associated antigens (Fig. 1-Active T cell mediated tumor killing). Under normal circumstances, once the antigen is eliminated, most effector T cells undergo apoptosis while a small fraction differentiate into memory T cells. However, in cancer, persistent stimulation of CD8 + T cells in tumor microenvironment results in a hyporesponsive state of T cell known as T cell exhaustion. T cell exhaustion shows progressive loss of effector function like loss of IL-2, TNF-α, and IFN-γ production and sustained expression of inhibitory receptors. High expression of inhibitory receptors such as PD-1, CTLA-4, Tim-3, LAG-3, B- and T-lymphocyte attenuator (BTLA), T-cell immunoreceptor with Ig and ITIM domains (TIGIT), NK cell receptor 2B4, and the glycoprotein CD160 are associated with the severity of the dysfunctional T cell phenotype. Reactivation of CD8 + T cells therefore presents a huge opportunity to target advanced tumors. Indeed, immune checkpoint blockers (ICBs) emerged as most promising therapeutic intervention in many types of cancer. At present, all the cancer immunotherapeutic approaches are based on the objectives of sustaining the activation of T-cells, and stimulating T-cell infiltration within the tumor [155] Fig. 2K. In the recent review, Waldman et al. has defined various T cell-based targeting approaches in cancer therapy [156]. The major immunotherapeutic strategies employed currently to attain these objectives include immune checkpoint blockers (ICBs), chimeric antigen-receptor (CAR)-based therapies, and cancer vaccines. In the following sections, the above-mentioned major cancer immunotherapeutic approaches have been described in detail.

## Cancer immunotherapeutic approaches

### Immune checkpoint blockers

Drugs targeting immune checkpoints, or their associated ligands have emerged as one of the most successful cancer immunotherapeutic approaches in several cancer types such as melanoma, non-small cell lung cancer (NSCLC), microsatellite instability high colorectal cancer, gastric cancer etc. Immune cell populations like T-cells, B-cells and cancer cells express repressor proteins, which when activated marks the termination of the immune response. These checkpoint proteins help in maintaining homeostasis of the immune response by controlling hyper immune activity to prevent autoimmunity. The major checkpoint proteins expressed by immune cells are programmed cell death protein 1 (PD1) and cytotoxic T lymphocyte-associated protein-4 (CTLA4). Recent studies have reported a few other checkpoint proteins, which include VISTA, TIM3, TIGIT, and LAG3. T- cell exhaustion is associated with high expression of checkpoint protein which inhibits T-cells clonal expansion and inactivates T cell immune response (Fig. 1—Immune evasion). Additionally, cancer cells also express high levels of checkpoint protein as well as its ligands (Fig. 1). TME containing high levels of exhausted T cells expressing checkpoint proteins benefits most from CPBs. Mostly, a three-factor metric system is used to predict the efficacy of immune checkpoint inhibitors. These factors include 1) expression levels of checkpoint proteins and their ligands, 2) tumor mutation burden, and 3) presence of CD8 + T cells within the tumor. However, these factors cannot be considered as the gold standard to ensure the therapeutic response, because primary resistance can strongly limit the efficacy of immune checkpoint therapy in a majority of cancer patients.

### T-cell based therapeutic strategies

Adoptive T-cell therapy is based on the principle of expanding the patient’s T-cell pool to enhance the immune system’s ability to identify and destroy tumor cells. Adoptive cell therapy involves isolation of autologous or allogenic T-cells from the patient or donor respectively, followed by ex vivo expansion and injection into the patient. Adoptive cell therapy has become a potential therapeutic option in many types of cancer [157, 158]. Infusing tumor-infiltrating lymphocytes (TIL) along with interleukins like IL-2 and lympho-conditioning showed promising results in a subset of patients with metastatic melanoma [159]. However, major disadvantages associated with these therapies include difficulty in predicting which patients will respond, and the cost and time involved in ex vivo production of T lymphocytes [160]. Recently, Morotti et al. has described in detail the promises and challenges of adoptive T cell therapy [161].

CAR-T cell therapy involves production of genetically engineered T cells expressing synthetic T-cell receptors (TCRs) specific to the tumor antigens. CAR-T cells have been proved to be very impactful cancer therapy in most of the relapsed or refractory hematological cancers. Several clinical trials involving CAR-T cell therapies have shown promising results [157]. The astonishing outcomes of these trials has resulted in the approval of several drugs by the European Commission (EC) and U. S. Food and Drug Association (FDA) between 2017 and early 2021 [162]. The major drawback associated with CAR-T cell therapy is the development of systemic inflammatory toxicity in the host body [163, 164]. Further studies are ongoing, which aim to reduce the bottlenecks associated with this line of cancer therapy. Fesnak et al. has provided a detailed review on the promises and challenges associated with CAR-T cell therapy [165].

Alternatively, CAR-NK cellular immunotherapy has been developed to overcome the CAR T-cells therapy limitations like graft-versus-host disease (GVHD), cytokine release syndrome (CRS), neurotoxicity etc., [166, 167]. Abundant availability from various sources and no HLA-matching restriction are the two major advantages associated with CAR engineered NK cells [168]. In contrast to CAR T-cells, CAR-NK cells are considered as safer because they may rarely cause GVHD, CRS and neurotoxicity. Additionally, CAR-NK cells secrete a totally different set of cytokines (e.g., GM-CSF and INF-γ) as compared to those pro-inflammatory released by CAR T-cells (e.g., TNF-α, IL-2, IL-6) [169]. Moreover, CAR-NK cells can identify and neutralize tumor cells both in CAR-dependent as well as CAR-independent manner [167]. CAR-NK cells pro-tumor effects are contributed by their ability to release of immunosuppressive cytokines (e.g., TGF-β, adenosine and indoleamine 2,3-dioxygenase) within immuno-suppressive TME, and also to express inhibitory receptors (e.g., TIGIT, PD-1, CTLA-1, NKG2A, CISH) [170, 171]. Therefore, the future efforts should aim at increasing the efficacy of the CAR-NK cell immunotherapy. Recently, a clinical trial (NCT03056339) was conducted on 11 patients to target CD19-expressing B-cell malignancies through CAR-NK cells derived from umbilical cord blood (UCB) [59]. 8/11 patients in this trial showed clinical response, and seven of those had rapid response and complete remission [172]. Altogether, the results from this trial and other ongoing clinical trials suggest that the CAR-NK cell therapy may represent the future opportunity in cancer immunotherapy. In the review by Albinger et al., an extensive comparative study describing the potential, limitations and ongoing clinical trials of CAR-based therapies i.e., the CAR-T and CAR-NK, has been described extensively.

### Cancer vaccines

Therapeutic cancer vaccines are personalized to patients and are based on the principle of activating host T cells by exposing them to tumor-specific neo-antigens (i.e., mutated proteins on cancer cells). The major hurdle to this therapeutic strategy is to identify and obtain the tumor-specific neoantigens. In several studies, cancer cells obtained from biopsies or whole cell lysate were used as vaccines, but in most of the cases it had failed to activate the host immunity-possibly due to an insufficient amount of tumor-specific neo-antigens. Hence, other approaches using dendritic cells are employed, in which host DCs are activated by tumor-specific neo-antigens, primed, expanded and injected back in the host circulation [173]. One such vaccine sipuleucel-T showed some initial success and was ultimately approved by the FDA in 2010 [174, 175]. mRNA vaccines are recently emerging as a mode to express neo-antigen peptides. However, these strategies are far from getting into the treatment regimens due to the complications associated with the production and administration [23]. Recently, Hu et al. in their review has provided a detailed account of the status of cancer vaccines in cancer therapy [176].

Despite the promising response observed with cancer immunotherapies, a large section of patients does not respond to the initial treatment or develop resistance later during the treatment. Several possible intrinsic and extrinsic factors contribute to such primary or acquired resistance. Aberrant intracellular signaling pathways in tumor cells is one of the key factor which inhibits T cell function and infiltration in the TME. One of the major pathways reported to be overactive in many cancer types, is the MAPK/ERK pathway which results in over-production of VEGF and IL-8, and establishment of an immunosuppressive environment for the T-cells [177]. Genetic mutations in EGFR and loss of function mutation in PTEN can activate the MAPK or PI3K pathways, which are reported to cause resistance to immune checkpoint therapy [178]. Genetic alterations in interferon-γ (IFN-γ), a prominent anti-tumor factor secreted by T effector cells, can also lead to immunotherapy resistance [179]. Aberrant β/Wnt signaling is also associated with primary or acquired resistance in many cancer types [180]. Furthermore, tumor cells are proficient in hiding from cytotoxic T-cells either by 1) preventing exposure of tumor-specific antigens on major antigen presenting cells by altering the MHC, or 2) by extensive trimming or modification of the antigen to be recognized by the T cells which lead to primary or acquired resistance. Furthermore, overexpression of immune checkpoint proteins and their corresponding ligands in the TME is a characteristic feature of many cancer types and is associated with T-cell exhaustion. This is one of the prominent factors involved in acquiring resistance against immunotherapy.

The extrinsic factors that contribute to primary or acquired resistance to immunotherapy include the components of the TME other than tumor cells. Myeloid lineage cells in the TME including tumor-associated macrophages, neutrophils, monocytes etc., inhibit T cells trafficking in the tumor, and sustain tolerance towards immunotherapy. Tregs, professional checkpoint cells, control T cells activity by secreting inhibitory cytokines or direct killing. High recruitment of Tregs in TME is associated with immunotherapy resistance [181]. TME’s acellular components such as hypoxia, ECM, low pH etc., also creates a highly hostile nutrient depleted, low oxygenated, acidic niche which favors T cell depletion or inactivation, thus playing a crucial role in acquiring resistance to various immunotherapies. Sharma et al. in their latest review provides a comprehensive account of the mechanism involved in immunotherapy resistance [2].

Highly efficient preclinical models are therefore urgently warranted to understand the complexity of the TME and it’s possible reprogramming for therapeutic interventions and the mechanism associated with therapy resistance.

## Models to study interactions of the TME

Several attempts have been made towards in vivo and in vitro modelling of the TME interactions, and most of these studies are based on 2D co-cultures, xenografts, humanized mice, and syngeneic mouse models [5, 182, 183]. Undoubtedly, 2D models offers a cost-effective model system with high degree of reproducibility of the conditions. However, the cells in 2D co-cultures form a monolayer, and thus the models are unable to accurately mimic the TME’s complex cellular interactions and signaling pathways [30, 184, 185]. Furthermore, the 2D models are unable to maintain the original morphology and polarization of the TME components. On the other hand, animal model systems are very expensive. Also, very often these in vivo model systems lack true representations of human-specific immune response [186, 187]. This partially explains why only 27% of the drugs with high efficacy in animal models can successfully reach to phase II clinical trials [188, 189]. Bioengineered 3D in vitro model systems can overcome several of the abovementioned challenges posed by 2D and animal models [182]. In the following sections, various 3D in vitro models and their utility in studying complex interactions within the TME will be described.

### Cell/extracellular matrix-based 3D models

In concordance with the fact that ECM is a major component of the TME with tumor-inducing capabilities, several cell/ECM-based 3D models have been devised to study interactions of the TME components [182].

Tumor cells are cultured within decellularized native tissues or natural/synthetic biomaterial scaffolds that provide proper cell adhesion, differentiation, and migration properties, and closely mimic the cell-ECM interactions [190–199]. While synthetic polymer provides higher degree of control and modulation of scaffold’s properties, the natural or decellularized ECM (dECM) recreate biochemical and structural environments similar to that of in vivo conditions by maintaining ECM and tissue-specific architecture [198, 199]. dECM have an additional advantage over other scaffolds as it considers ECM environment as a whole, whereas scaffolds focus on specific ECM components. The major concern with dECM-based 3D models is the intactness of the tissues, as decellularization procedure involves treatment with enzymes and detergents [200]. Along with selection of scaffolds biomaterials, cell types and physical/chemical conditions are other critical factors that determine the experimental outcomes of tumor modeling. For instance, seeding porous scaffolds with different cell populations of the TME, and allowing them to proliferate and rearrange, result in a 3D matured scaffold culture condition. Whereas cells cultured in a hydrogel-based scaffolds, after proliferation and undergoing rearrangement generate a matured cell-laden hydrogel. Further, depending on the hydrogel and cell type used for seeding, such matured cell-laden hydrogels can form cell clusters/organoids. While maintaining the original hydrogel network, matured and rearranged cell populations can also produce ECM in such in vitro 3D models. The hydrogel-based model’s mechanical properties can be modulated in order to closely mimic tumor ECM [182].

Based on these considerations, Rijal et al. developed a native ECM based tissue matrix scaffold (TMS) [201]. This in vitro model consists of multilayered tissue culture platform derived from mouse mammary tissue. Culturing of the stromal and cancer cells in compartmentalized manner, induces the expression of extracellular and intracellular biomarkers of breast cancer, thus confirming the correct proliferation and cancer growth. Therefore, this model mimics mammary tissue, and can be used for specific tumor biomarkers screening [201]. Similarly, Hume et al. created a 3D tumor model by culturing tumor cells and adipocytes in collagen scaffolds [197]. Presence of adipocytes was observed to promote cancer cell invasion by increasing tumor cells migration, while decreasing the total number of drifting cells. Thus, this 3D tumor model clearly demonstrates the heterogeneous cell behavior within TME [197].

Scaffolds can also be used to develop more recent in vitro 3D bioprinted models, and investigate spatio-temporal patterning of cells in tumor [187]. In 3D bioprinting technique, bioinks (sorted individual cell types and/or scaffold/hydrogel) are deposited in a predefined manner using a 3D bioprinter and then crosslinked to the carrier material (scaffold/hydrogel) to generate a stable culture. Depending on the bioinks and the specific aim of the study, different types of mature 3D bioprinted models can be obtained after cells proliferation and rearrangement, i.e., scaffold-free culture, semi-scaffold free culture and scaffold-based cultures. Such 3D in vitro model’s composition and architecture is well-defined with higher degree of reproducibility. These model systems have enabled us to introduce tumor cells into the complex TME, and to study ECM deposition, interaction of different TME cell types, and self-organization of the tissue itself [202]. Recently, Langer et al. has bioengineered a more desmoplastic TME by sequentially incorporating breast cancer cell core surrounded by human mammary fibroblasts, umbilical vein endothelial cells, primary human preadipocytes and mesenchymal stem cells [202]. Using this 3D tumor model, self-organization of tissue, interaction of tumor and stromal cells, and ECM deposition in complex tumor microenvironment could be learned [202].

Bioprinted 3D in vitro models can also be used to investigate time-dependent studies such as the kinetics or dynamics of administered drugs, growth factors and metastatic dissemination of cancer cells over time [203]. For instance, Meng et al. bioprinted 3D model to recreate TME that primes metastatic spread of lung cancer [203]. Taken together, with the advent of 3D bioprinting technique, creation of spatially defined 3D in vitro models has been improved. Well-defined composition and architecture, provision to use variety of materials with higher precision while fabrication, and commercial availability has further enhanced the utility of these 3D model systems. The common limitations associated with development and utilization of such in vitro 3D models include toxicity of bioinks, slow printing speeds and lack of reproducibility to devise state-of-the-art model system [204, 205].

### Cell-based 3D models

In cell-based 3D models, the cells are present as aggregates, and usually they formulate their own intrinsic ECM. Thus, these models closely mimic tumorigenesis, the physiological framework of tumor organization, and tumor-stromal interactions within the TME [182]. Depending upon the differences in the initial stages of constitution, cell-based 3D models can be broadly classified as 3D (hetero)spheroids and organoids.

#### 3D (hetero)spheroids model system

3D (hetero)spheroids are the most extensively used 3D model system in cancer biology and are specifically used for anticancer drugs testing. Such spheroid models are formed as a result of forced aggregation of selected cells that manifest high deposition of ECM or high cell–cell contact [182]. The main advantage of using cell-based 3D (hetero)spheroids model systems is its ability to precisely recreate important in vivo tumor microenvironment features such as morphology constituted by multiple cell layers, cell–cell/cell-ECM interactions, cellular heterogeneity, gene expression patterns, and cell signaling pathways [206]. Moreover, these 3D (hetero)spheroids provide flexibility to maintain different types of physical/chemical gradients and integrate multiple cell types [182, 206]. In conjunction with 3D bioprinting technologies, 3D (hetero)spheroids are cost-effective 3D culture method with an ability to create more physiologically representative tumor model. Although the cell-based 3D (hetero)spheroids model systems have immensely advanced our cancer biology knowledge, there are few constraints associated to it such as slow fabrication rate and more costly as compared to 2D cultures [182]. Furthermore, lack of homogeneity in the size/morphology of cell aggregates in spheroids compromise the reproducibility, and thus impedes the development efforts of the cell-based 3D standard models [207].

#### Organoids- 3D tumor model system

Organoids are 3D tumor models generated as a result of proliferation and self-organization of a single progenitor cell. Therefore, these models can closely mimic the architecture and the complexity of the tissue of origin [208]. Unlike spheroids, organoids are developed based on the genetic programming of the progenitor cell, thus mimicking the actual tumor development more closely. The major advantage of organoid 3D models is their distinct capability to follow different stages of native tumor progression trajectory, and therefore its capability of retaining the cellular heterogeneity and maintaining the pathophysiology of the tumors in vitro [209]. Moreover, as organoids can retain salient tumor features in three-dimensional space, these models are suitable for studying tumor-stroma interactions. However, organoids has some limitations such as its development is time-consuming with high degree of variability between experiments, some of the mature organoid models does not truly represent in vivo conditions, and lack stroma and vasculature system [210]. Furthermore, the tumor organoid models lack immune-competent microenvironment and stromal components because of the epithelial origin of the progenitor cells. This drawback has been overcome by co-culturing organoids with stromal and patient’s immune cells [211, 212]. More advanced organoid culture systems with well-defined architecture, cellular composition and signaling profiles can be developed by combining 3D bioprinting technique with organoids [211, 213]. Such combined strategy has the potential to ensure both precise spatial arrangement of cells in 3D models and maintaining the hierarchical-like architecture of the TME [182].

3D organoid models developed using patient-derived tissues are known as patient-derived organoids (PDOs). These tumor model systems have removed the bottleneck of extrapolation of results from animal and patient-derived xenografts (PDX) models [210, 211]. PDOs can be generated from various types of adult stem cells (ASCs) and pluripotent stem cells (PSCs) from distinct tissues through a procedure like human organogenesis [214, 215]. PDOs derived from ASCs contain epithelial cells and are suitable model system to study tissue regeneration and homeostasis. Contrastingly, PSCs derived PDOs can contain cells of both epithelial and non-epithelial origins, and are appropriate to study organ development [216]. Stability of gene expression profiles and reproducibility of model system are the major advantages associated with the usage of PDOs [217]. PDOs recreates basic characteristics of primary tumors while maintaining genomic and transcriptomic profiles of primary tumors [209, 218–226]. The heterogeneities specific to patients and cancer subtypes can be captured using individual PDOs, which has been reported to be in more consent with the actual patients response to drugs [227]. Additionally, PDOs can also be very useful in developing patient-specific treatment strategies. By combining organoids derived from healthy and tumor tissues from a patient, the efficacy of large number of drugs can be tested at the same time. The best drug for a patient responds by selectively killing tumor cells without damaging the healthy ones [227]. Moreover, the ease of manipulation of PDOs through CRISPER/Cas9 approach has encouraged its implementation for tumor modelling and identifying significant driver mutations involved in the tumor development and progression [228]. Recently, circulating tumor cells (CTCs) isolated through non-invasive liquid biopsies of cancer patients has also been used to develop PDOs. These CTC-derived organoids could be pivotal in gaining genetic and epigenetics insights about cancers in patient-specific manner [229]. Collectively, the development of PDOs has enormously transformed the drug and target discovery research arena, providing new avenues for drug testing, and designing personalized therapeutic interventions in a pre-clinical setting [230].

While PDOs models have many advantages, there are several limitations associated to it. These cell-based 3D models have been reported mostly to be deficient of essential components like blood vessels, stromal cells, immune cells, surrounding mesenchyme and neurons, thus lacking the typical TME [208]. Deficiency of immune cells within PDOs TME appears to limit its value in studies evolving tumor immunotherapy approaches [227]. For instance, at present, the efficacy determination of the inhibitors of immune checkpoints programmed death-1/programmed death ligand-1 (PD-1/PD-L1) using PDOs model cannot be conclusive and need further research attention [231]. Recently, there have been efforts to create a native immune microenvironment within PDOs by co-culturing of more complex immune and stromal components in a more compartmentalized manner [232, 233].

In summary, PDOs are good substitute model for understanding tumor biology, drug screening, and development of therapeutic approaches because of the aforesaid advantages. Currently, research involving the development of PDOs and its clinical applications are still in its early stage. In future, PDOs-oriented research should focus on the improvement of TME by overcoming present barriers and conducting more clinical trials on PDOs model systems with precisely defined composition and architecture.

### Microfluidics models

Development of microfluidic systems, and their application in building organ-on-chip devices (platforms that can model physiological functions of tissues and organs) have revolutionized the field of tumor biology [182]. The main advantage of these in vitro 3D culture models is their flexibility to modulate various parameters independently. In these models, optimized cell survival conditions can be achieved by maintaining desired cellular heterogeneity and localization, chemical gradients, tissue interfaces orientations and mechanical forces [234]. Such tumor biology-specific microfluidic cancer-on-a-chip (CoC) models are now becoming preferred systems due to their microscale volume requirement which makes it cost-effective as compared to other 3D culture protocols and bioreactors [234–238]. Moreover, heterotypic cancer-on-a-chip 3D model systems generated by culturing multiple cell types in a dynamic microenvironment of the microfluidic chip can be used to understand distinct interactions/communications between tumor cells and various TME cellular and acellular components [239]. For instance, T-cell infiltration rates within TME has been studied on a heterotypic 3D microfluidic platform developed from breast cancer cell, human umbilical vein epithelial cells (HUVECs) and monocytes confined spatially in a gelatin hydrogel, and T cells dispersed in the medium [239]. The results showed higher rates of T-cells infiltration in presence of monocytes in the medium as well as extreme hypoxic conditions stimulated by using tumor spheroids instead of diffused cancer cells [239]. Furthermore, the effects of different growth factors (GFs) or drugs in a biomimetic microenvironment and stroma-driven ECM remodeling can also be studied using these microfluidic chip-based in vitro 3D models with a high degree of monitoring [240, 241]. Carvalho et al. have developed a microfluidic chip-based in vitro 3D model to mimic human colorectal cancer TME’s microvascular tissue functions. This model system allowed radial drug access into solid tumors and was used to assess the dynamic interaction between endothelial cells and colorectal tumor cells in a time-dependent manner [240]. Additionally, Gioiella et al. made a tumor-on-a-chip model with a stromal compartment to investigate tumor-stroma activation-dependent ECM remodeling [241]. Recently, innovative 3D in vitro models have been developed by combining the features of tumor spheroids/organoids and microfluidic chip systems. These models can be used to study tumor-stroma interactions and their systemic effects simultaneously [27, 242, 243]. For example, a 3D microvascular network of endothelial cells generated in two distinct microenvironments i.e., bone and muscle with osteo-differentiated cells and smooth muscle mesenchymal stromal cells (MSCs) respectively, showed different tumor cell extravasation rates, and thus confirming the TME’s role in cancer progression [244]. Moreover, in contrast to inadequately aligned endothelial cells grown in 2D culture system, cells in this model manifested phenotypes similar to in vivo conditions.

Although these microfluidic models have enabled us to define the different aspects of TME, there are certain limitations associated to its usage. Microfluidic model system needs to be more robust and reliable such that they should not be affected by any external or internal impairments like air bubbles, hindered laminar flow etc., [245]. Moreover, development of a new chip fabrication material is urgently needed, as polydimethylsiloxane (PDMS), the most commonly used material, can retain small molecules non-specifically [27, 246]. Taken together, these microfluidic systems in combination with other 3D in vitro tumor models and emerging integration/fabrication technologies have potential to reveal the tumor-stromal interactions at higher resolutions.

In conclusion, 3D culture model systems are successful up to a certain extent in reconstituting the complex TME in vitro*.* However, they are still in their early stage of development, and therefore, limitations must be addressed appropriately before deriving any conclusions. One of the most critical features to consider in clinical tumor samples is the patient heterogeneity contributed by patient-specific tumor burden, immune cell types within TME, and the tumor stroma content. The incorporation of all these variables in in vitro models is paramount, however, it makes the model complex, and may compromise the reproducibility. Moreover, at present, the development of novel culture methods/protocols that allows long term in vitro maintenance of different cell sub-populations, and integration of multiple cell types in a single model is urgently required. Additionally, research should also be focused on the development of strategies required for the inclusion and continuous renewal of the diverse immune cell populations within 3D models. Along with overcoming challenges at experimentation front, the advanced in silico approaches can partially complement the pace of understanding different underlying features of the TME and defining its therapeutic potential. In the following sections, different aspects of TME profiling that can be achieved using state-of-the-art computational approaches have been described.

## In silico approaches of TME profiling

In the backdrop of biological significance, the extent of the success of cancer immunotherapy approaches highly relies on the intricate interplay between tumor cells and TME’s immune and stromal components. Detailed molecular- and cellular-level characterization of this dynamic ecosystem can delineate strategies for designing more effective therapeutic interventions and identify novel biomarkers capable of classifying therapy responders and non-responders [247]. With the advent of high-throughput technologies, it is now possible to study TME complexities experimentally at the genomics, epigenomics, transcriptomics and proteomics levels at resolutions ranging from whole organisms to single cells. However, most of these assays require dissociation of the tumor tissues, which in turn can modify cells phenotypic and population representations [248, 249]. In order to overcome the above-mentioned challenges, computational methods have been devised. These in silico methods have helped us to understand the complexities of the TME and derive inferences from bulk tissue gene expression profiles. In the following sections, we will discuss various in silico analysis tools developed to determine tumor purity, immune repertoire profiling, and neoantigen predictions. We will also describe the computational methodology and models developed to screen prognostic genes in the TME.

### Tumor purity and TME immune cell types profiling

Computational approaches to determine tumor tissue composition can be broadly classified into two categories, namely enrichment methods and deconvolution methods [250]. The success of both these classes of approaches depends on the prior acquaintance of the marker genes with the cell types of interest. The enrichment strategies aim at identifying tissue-specific differentially expressed gene sets or pathways that depict distinct cell populations [251]. However, such methods are unable to compute proportions of discrete cell types and cannot differentiate between cell subtypes with common gene markers [250]. In contrast, deconvolution methods can perform in silico evaluation of the proportions of distinct cell types along with closely related cell subpopulations. Deconvolution strategies can also overlay gene expression data from bulk tissue transcriptomes to specific cell types [249, 252–254]. Additionally, ATAC-seq and DNA methylation profiles can be used to evaluate tumor tissue compositions [255, 256]. In the recent years, the combination of automated tissue dissection protocols and scRNA-seq data has emerged as the preferred methodology to explore novel cell sates in bulk tissues [247].

Identification of diverse immune cell types in tumor based on precise signatures is an urgent task. The presence of hierarchical sub-clonal populations, and differences in the TME background composition with that of normal cells pose additional intrinsic complexities [257]. However, a deeper understanding of the immune cells repertoire in TME both qualitative and quantitative is essential for designing successful therapeutic interventions. Similar to TME composition’s delineating computational efforts, several tools have been developed specifically for estimating relative proportions of different immune cell types and subtypes within a sample using their specific gene expression profiles [254, 258]. Details of the bioinformatics tools commonly used to assess tumor purity, estimate stromal or immune fractions from bulk tumor transcriptomes, and identify immune cell types are tabulated in Table 1.

**Table 1:** Bioinformatics tools developed to assess tumor purity, compute cell proportions, and identifying specific cell-type subsets

In silico tools for determining tissue composition	Description	References
UNDO	Identify cell type-specific marker genes, compute sample-wise cellular proportions, and deconvolute mixed expressions into cell-specific expression profiles	[306]
contamDE	Estimate cell proportions and perform differential gene expression analysis from RNA-seq data considering tumor-infiltrating normal cells as contaminants	[260]
ISOpureR	Cancer cells fraction estimation, and personalized patient-specific mRNA abundance profiling from a mixed tumor profile	[261]
ISOLATE	Primary site of origin prediction, sample heterogeneity effect removal and deconvolution, and determination of differentially expressed genes of tumor purity	[307]
ESTIMATE	Gene set enrichment analysis method that uses expression profile of immune, stromal, and tumor cells signature genes to give tumor purity scores	[259]
DeMix	Maximum likelihood-based statistical approach for computing cell fractions, and differential gene expression analysis of tumor purity	[263]
PurBayes	Bayesian statistics modelling approach that uses RNAseq data to estimate sub-clonality and tumor purity	[265]
DeconRNASeq	Deconvolution of heterogeneous tissues using mRNA-seq data. Estimates proportions of distinct immune cell subsets	[308]
PSEA	Computes cell fractions from marker genes expression profiles	[309]
csSAM	Differential gene expression analysis using microarray data for each cell type in the sample and their relative frequencies of occurrence	[254]
NMF	Computes cell-type-specific expression profiles and their proportions without any a-priori information	[310]
DSA	Probabilistic model-based approach that uses RNA-seq data from heterogeneous samples to estimate cell-type-specific transcript abundances	[311]
MMAD	Simultaneous calculation of cell proportions and cell-specific expression profiles; prior knowledge of cell fractions and reference expression profiles are required	[312]
PERT	Probabilistic gene expression deconvolution strategy that corrects perturbations in reference expression profiles of different cell populations of a heterogeneous sample	[313]
LLSR	Computes different cells proportions from reference microarray expression profiles	[314]
CIBERSORT	Estimates cell proportions from complex tissues using their gene expression profiles	[271]
Nanodissection	Computes gene expression profiles of specific cells/tissues using reference expression profiles as training data for this genome-scale machine-learning based approach	[269]
Dsection	Probabilistic model using reference expression profiles and predicted cell proportions information. Estimate cell proportions and cell-specific expression profiles with better accuracy	[268]
MCP-counter	Estimates abundance of two stromal and eight immune cell types of populations in bulk tissues	[251]
EPIC	Computes absolute fractions of tumor and different immune cell types using transcriptomic data	[315]
xCell	Infers abundance of 64 stromal and immune cell types based on cell-specific gene signatures enrichment	[316]
TIMER	Six immune cell-types infiltration quantification across different cancer types based on RNA-seq data	[317]
MethylCIBERSORT	CIBERSORT-based deconvolution method. Uses DNA methylation data from bulk to infer tumor cell fractions	[318]
DeMixT	Extract component-specific proportions and gene expression profiles for every sample	[252]
MuSiC	Single cell RNA sequencing data derived cell type specific expression profiles are used to define cell compositions from bulk RNA sequencing data in complex tissues	[319]
CPM	Deconvolution algorithm that uses single cell RNA sequencing reference expression profiles to infer cellular heterogeneity in complex tissues from bulk transcriptome data	[320]
CIBERSORTx	Estimates sample-wise cell type frequencies from bulk RNA sequencing data using single cell RNA sequencing or bulk-sorted gene expression reference profiles data, and minimizes platform-specific variations	[249]
quanTIseq	Using bulk RNA sequencing data, this method quantitates proportions of 10 types of immune cells	[321]

Few of the tools listed in Table 1 have already been used to discover potential prognostic and therapeutic biomarkers [259–261]. ESTIMATE in particular, has emerged as one of the widely used method, and is currently employed in several standard analysis pipelines of The Cancer Genome Atlas (TCGA). It determines the general fractions of stromal and immune components of the tumor based on stromal and immune scores derived from the gene set enrichment analysis (GSEA) of the stromal and tumor signature genes. Integration of immune and stromal scores resulted in an estimate score that defines the tumor purity in a sample. Recently, four distinct consensus molecular subtypes (CMSs) of colorectal cancer has been identified based on the immune subtype signatures characterization using ESTIMATE [262]. These subtypes have distinguishing features: CMS1 (14%) highly mutated, strong immune activation, microsatellite unstable; CMS2 (37%) epithelial origin, marked activation of MYC and WNT signaling pathways; CMS3 (13%) epithelial origin, highly dysregulated metabolic pathways; CMS4 (23%) mesenchymal origin, marked TGF-β activation, highly aggressive stromal invasion and angiogenesis. Approximately 13% of colorectal cancer samples in the study cohort showed features of multiple consensus molecular subtypes, which can be probably linked to intra-tumoral heterogeneity or transition phenotypes. DeMix, a linear model-based tool, computes the proportion of stromal and tumor cells in samples by considering the transcripts contributed by the epithelial and stromal component of a tumor sample [263]. As an input, this tool requires gene expression profile of at least one gene of each cell type. Recently, the characterization of heterogeneity among 333 primary prostate carcinomas samples has been performed using DeMix, and has led to the identification molecular targets with therapeutic potential [264]. Moreover, the Bayesian statistics-based PurBayes tool evaluates the tumor purity and sub-clonality of tumor samples by utilizing the expression data of tumor, stroma and matched normal signature genes [265].

Initial methods devised to estimate the relative fractions of distinct immune cell types within the tumor samples require some degree of prior information regarding defined cell types, their relative proportion, and specific gene signatures with or without expression profiles [257]. However, the recent developments utilize data mining approaches, and aims at minimizing this dependency of prior knowledge by defining distinct immune cell types based on marker genes [263, 266, 267]. csSAM and Dsection are two popular bioinformatics approaches that capitalizes on these recent developments [254, 268]. csSAM, a linear regression model, uses information of known cell proportions in the sample to determine the cell-specific expression profiles. Using csSAM, the comparative gene expression profiling of whole-blood from patients with stable kidney transplant and those experiencing acute rejection has revealed several hundreds of differentially expressed genes that has remained undetectable previously [254]. Dsection, an implementation of a probabilistic approach, determines cell type proportions in heterogeneous tissue samples, and differential cell-specific expression patterns under various experimental conditions by using previously estimated cell proportions and their reference expression profiles [268]. Nanodissection is a supervised machine learning-based iterative framework that identifies cell/tissue specific transcripts within the sample. For model training purpose, nanodissection method require a small set of marker genes and the reference expression profiles [269]. Quigley et al. has used this algorithm to identify cell type-specific transcripts, and used them successfully to examine the presence of cytotoxic T-lymphocytes, T-helper 1, T-helper 2 and B cells in the breast tissue. This study also shows higher cytotoxic T-lymphocytes infiltration in integrative cluster 10 (IC10)/basal-like breast cancers with wild type TP53 mutation, thus suggesting association between inactivation of TP53 and tumor immunosurveillance failure [270]. CIBERSORT is another popular supervised machine learning algorithm used to quantify immune cell types in a highly heterogeneous transcriptome samples. As an input, this tool requires precisely defined signature genes specific to various immune cell types populations and their proportions [271]. In clinical setting, CIBERSORT has been recently used to identify leukocyte diversity and prognostic genes within and across 25 tumor types from TCGA database [270]. In addition, Gentles et al. applied CIBERSORT on bulk transcriptomics data from 40,000 tumors to investigate immune and tumor heterogeneity, and identified intricate correlation between 22 leukocyte cell types and survival outcomes [272]. In another study, Thorsson et al. integrated tumor-infiltrating lymphocytes genomics, hematoxylin & eosin imaging data, CIBERSORT deconvoluted transcriptomic data of immune cell fractions, TCR & BCR repertoire, neoantigen prediction, expression of immune gene, somatic DNA alterations, and viral RNA expression. By implementing this multi-omics approach, six immune cell types shared across multiple tumor categories were identified [11]. An implementation of CIBERSORT, i.e. MethylCIBERSORT uses DNA methylation data from bulk tumors, deconvolution estimates and hot/cold tumors data from TCGA to define composition of tumor tissues. Singh et al. has identified distinct tumor clusters based on immune cell proportions in glioblastomas. In contrast to isocitrate dehydrogenase (IDH) wild type glioblastoma cases, where five optimum clusters were recognized based on immune cell types proportions, the IDH mutant glioblastoma samples have only two optimal consensus clusters [273]. Also, in IDH wild-type glioblastomas, tumor clusters were found to be associated with oncogenic alterations like CDKN2A/B deletion and EGFR amplification [273]. Similarly, chromatin accessibility profiles (estimated using ATAC-seq protocol) of tumors has also been used to define 16 major cell types of normal hematopoietic and leukemic hierarchies in human blood from 12 acute myeloid leukemia (AML) and 9 healthy humans [255]. Taken together, the advancements to determine tumor purity and immune cell heterogeneity within TME has contributed immensely to our present understanding of both tumor and immune biology.

### Screening of TME-related prognostic genes

The identification of prognostic TME-related genes for predicting outcomes has enormous potential [274, 275]. The first step towards building a prognostic model is the acquisition of gene expression and clinical data for the given cancer type. Single-sample gene set enrichment analysis for each sample in the cohort generates stromal and immune scores. Patients are then grouped as high/low stromal and high/low immune subgroups based on the median values of the respective scores. For each of the stromal and immune score groups, differentially expressed genes are identified. Functional enrichment analysis of the intersecting differentially expressed genes across different score subgroups helps to elucidate the potential and significant biologic functions [275]. Furthermore, survival analysis is performed to analyze intersecting differentially expressed genes and their prognostic association with patient’s overall survival (OS). Protein–protein interaction (PPI) networks are constructed for differentially expressed genes with prognostic values, and central genes with higher degrees of connection are identified. Further univariate and multivariate regression analyses are performed to obtain the most significant prognostic value genetic signature, which in turn is used to establish a risk score formula for predictive purposes.

Recently, Chen et al. has systematically investigated pancreatic tumor microenvironment, and established biomarkers associated with tumor/stromal cell populations. Overall survival analysis showed that the high immune/stromal group of pancreatic patients are closely related with poor prognosis [274]. In this study, four signature genes COL2A1, CXCL10, TRPC7 and CUX2 emerged as independent prognostic factors. The prognostic model created using these signature genes assigned high risk scores to KRAS and TP53 mutations. Additionally, at single cell resolution, CXCR3 was found to be highly expressed on T cells, whereas its ligand CXCL10 is abundant on tumor associated macrophage population [274]. Similarly, Ye et al. performed thorough examination of breast cancer (BC) microenvironment, and identified three signature genes namely SIT1, KLRB1 and GZMM as prognostic factors [275]. All these three genes were found to be negatively correlated with tumor purity, and positively associated with the intrusion of immune cells like B cells, neutrophils, CD4 T cells, CD8 T cells, macrophages and dendritic cells in BC microenvironment [275]. In conjunction with the necessary experimental validation, these identified prognostic genes could be promising candidates for therapeutic interventions.

### Immune cell receptor profiling and neoantigen prediction

Immune cell repertoires i.e., T-cell receptors (TCRs) and B-cell receptors (BCRs), recognize and neutralize a highly diverse range of antigens [247]. Quantitative studies of these immune cells repertoires in various cellular compartments can be performed using high-throughput sequencing. Lymphocyte-specific TCR and BCR sequencing approaches have enabled the analysis and tracking of diverse lymphoid cell populations, which in turn has further increased our understanding of intra-tumoral, inter-tumoral and clinical outcomes heterogeneity. The ability to profile only a subset of cellular heterogeneity, and inability to discriminate between cellular states, i.e., naive vs. activated, are the two major shortcomings of these immune cell receptors profiling methods [247].

Immune repertoire sequencing has provided opportunity to study immune cell heterogeneity in the TME of various cancer types [276, 277]. For instance, TCR sequencing has been used routinely to understand the T cells repertoires after immune checkpoint blockade therapy. In melanoma patients, anti-CTLA-4 and anti-PD-1 therapies have been shown to increase the diversity of TCRs, and T cell clonotypes [278, 279]. The major challenge for tools analyzing immune cells repertoires include making a clear distinction between rare clones in bulk data, and identifying sequencing and/or PCR errors. Furthermore, recent technological advances have enabled simultaneous transcriptomics analysis and BCR/TCR profiling at single-cell resolution. Various bulk and single-cell repertoire analysis tools are listed in Table 2.

**Table 2:** Bioinformatics tools of immune cell repertoire analysis

Insilco tools of cell repertoire analysis	Description	References
Bulk cell repertoire analysis tools		
IGMT/V-QUEST	Analyze cell repertoire generated from rearrangement of nucleotide sequences of antigen receptors (immunoglobulin or antibody, and T cell receptors (TCRs))	[281]
IgBLAST	Perform sequence analysis of immunoglobulin’s variable domain	[280]
iHMMune-align	Hidden Markov model-based immunoglobulin heavy chain (IGH) gene characterization program that identifies germline genes in rearranged immunoglobulin sequences	[282]
MIGEC	Corrects PCR and sequencing errors from immune cell repertoires while maintaining the indigenous diversity	[322]
MiXCR	Quantitate clonotypes from large immunome sequencing data, identifies germline hypermutations, and corrects PCR/sequencing errors using heuristic multilayer clustering	[283]
TRUST	Detect tumor-infiltrating T cells by de novo assembly of hypervariable CDR3 sequences, and aligning it to sequence of reference genes from International Immunogenetics Information System (IGMT)	[323]
GLIPH	Estimates T-cell response diversity by grouping different TCRs sequences that can identify the same antigen-MHC complex	[285]
Single cell repertoire analysis tools		
TraCer	T-lymphocytes single cell RNA sequencing data is used to regenerate paired and full-length TCR sequences Transcriptional profiles based clonal relationships is used to link T-cell specificity with functional response	[287]
scTCR-seq	Using single cell TCR sequencing data, accurate identification and assembly of full-length T-cell receptor sequences	[324]
TRAPes	Algorithm uses paired end, short reads from single cell RNA-seq libraries to reconstruct TCR repertoire, and understand cell state heterogeneity	[325]
VDJPuzzle	Single cell RNA seq reads overlapping to VDJ or constant region of reference set are assembled using Trinity, filters with IgBlast to create new TCR reference set, and aligns against this new reference	[326]

Early methods of immune cell repertoire profiling include IgBLAST, IHMMUNE-ALIGN and IMGT/V-QUEST [280–282]. MiXCR is a more recent and widely used sequencing-based approach of bulk BCR and TCR profiling. This method is capable of correcting PCR errors, and identifying germline hypermutations by applying multilayer clustering algorithm [283]. Ma et al. analyzed the impact of functional DNA damage repair (DDR) gene polymerase epsilon (POLE) mutations on tumor immune microenvironment post immune checkpoint blockade (ICB) therapy [284]. The authors observed upregulation of immune-related pathways in post-ICB Pole^P286R^ tumors. Evaluation of the TCR-beta CDR3 clonotypes isolated through MiXCR showed a higher rate of clonal expansion, richness and decreased evenness in post-ICB Pole^P286R^ tumors [284]. Another method GLIPH, clusters TCRs based on their global similarity between CDR3 sequences, and the conserved motifs that provide common specificity to the receptors [285]. Subudhi et al. have shown that the autoreactive T cell clonal expansion and diversification of T cells repertoire occurs in prostate cancer patients treated with CTLA-4 blocking antibody or those experiencing immune-related adverse events (irAEs). This supports the hypothesis that newly established responsiveness to shared antigens may led to inflammatory response in cancer patients undergoing blockade therapy [286].

With the advent of single cell technologies, it is now possible to perform single-cell TCR analysis. Such analysis has enabled the pairing TCR of α and β chains sequences. TRACER tool generate all possible pair of TCR α and β chains by aligning all the possible V and J segments, then assembling the reads in contiguous sequences [287]. Zheng et al. implemented TRACER tool for TCR profiling of > 5 k single T cells isolated from blood, tumor and adjacent normal tissues of hepatocellular cancer patients. The coupled TCR and transcriptional profiles facilitated the identification of functionally 11 T cell subsets, and their developmental path [288]. Moreover, to understand the activity of cells and their correlation with antigen receptor sequences, new methods of RNA-seq and repertoire sequencing from the same cell has been developed.

In addition to TCR sequencing of T cells in the TME, the prediction of neoantigens from patient’s DNA or RNA represents a major step towards personalized therapeutic approaches [289]. Neoantigens are present only on tumor cells but not on normal cells; therefore, neoantigens can elicit tumor-specific immune responses. These mutation-associated cancer antigens are cleaved, and short peptides are presented to TCRs on MHC molecules. In view of the unprecedented possibilities of neoantigens, TCR rearrangements, and large variations in MHC molecules, there is an urgent need of neoantigens prediction tools [290]. Neoantigen prediction is a three-step process: identifying the mutation-associated cancer proteins, HLA typing, and determining neoantigen affinity towards MHC binding [290]. List of in silco tools and pipelines developed to analyze different steps of neoantigen prediction independently or combined are listed in Table 3.

**Table 3:** In silico tools and pipelines for Neoantigen predictions

Bioinformatics tools	Description	References
Identification of genome variant		
GATK	Genome analysis toolkit Identify variants across genome using next generation sequencing data	[292]
MuTect	Somatic point mutation identification in cancer genomes	[291]
HLA typing		
Polysolver	Three major MHC I genes alleles identification based on whole exome sequencing data	[294]
OptiType	HLA genotyping algorithm that predicts all major and minor HLA class I alleles from next generation sequencing data	[293]
MHC-binding affinity		
netMHC/netMHCII/netMHCpan/netMHCpanII	Prediction of MHC binding affinity to Class I and Class II MHC molecules	[298–300, 327]
SMM	Sequence specificity-based quantitative model to identify binding affinity to MHC I molecules	[328]
SMMPMBEC	An amino acid similarity matrix derived based on experimental peptide-MHC binding interactions Act as Bayesian prior for prediction of peptide-MHC class I complex interaction	[329]
MHCflurry	Allele-specific neural networks trained on MHC ligands identified by mass spectrometry and binding affinity measurements to develop a model for prediction of MHC I complex proteins and their ligands	[330]
EDGE	Deep learning approach of HLA prediction based on training data from 74 patients	[301]
Pipelines combining all steps of neoantigen prediction		
FRED 2	Prediction, selection, assembly and HLA typing of T-cell epitope	[302]
NetTepi	Predicts peptide-MHC (pMHC) binding affinity based on integration pMHC stability and T-cell propensity predictions	[304]
pVAC-Seq	Predicts tumor-specific neoantigen based on the integration of tumor mutation and expression data	[331]

MUTECT and GENOME ANALYSIS TOOLKIT (GATK) are two well-known variant analysis tools that implement Bayesian classifier principle to detect single nucleotide polymorphism [291, 292]. A limitation associated with such variant analysis tools is to determine the functional implications of a variant on different transcripts, so the choice of tools and database is critical. In the second step of neoantigen prediction, i.e. HLA typing, a sensitive assembly or mapping strategy is involved, which in turn relies on well-annotated reference genome. OptiType and PolySolver are examples of some of the popular HLA typing tools [293, 294]. Park et al. used the HLA-I genotype information of > 1500 patients suffering from 11 different cancer types from two independent studies using OptiType and PolySolver tools for prediction. The results revealed that HLA-I heterozygosity is positively correlated with early onset of tumor [295]. Finally, at the MHC-binding affinity estimation step the non-linear, machine-learning based methods such as NETMHC, NETMHCII have shown improved prediction accuracy as compared to those early sequence-based methods like SYFPEITHI and BIMAS [296–300]. EDGE, a neural network-based computational model for epitope prediction has been developed using HLA mass spectrometry neoantigen peptides and genomic data of 74 cancer patients [301]. In comparison to tumor sets binding-affinity features, EDGE have a nine-fold higher positive predictive value.

Bioinformatics analysis pipelines integrating the abovementioned multiple steps of neoantigen prediction are tabulated in Table 3. For instance, FRED 2 provides a unified immunoinformatics framework for T-cell epitope prediction, selection, assembly and HLA typing [302]. Loffler et al. implemented FRED2 pipeline for defining different aspects of tumor neoantigens in hepatocellular carcinoma, and concluded that the mutated HLA ligands derived from exome represent the limited targets for personalized immunotherapy approaches [303]. Therefore, an increase in the neoantigen search space is needed to identify potential targets and enhance the efficacy of tumor immunotherapy approaches, especially in case of malignancies with lower or similar mutational burden. NetTepi is another integrated approach of T cell epitope discovery that combine predictions of peptide-MHC (pMHC) binding affinity, stability and T cell propensity [304]. Recently, Buckley et al. identified NetTepi as one of the best peptide immunogenicity prediction tools in an unbiased comparative evaluation of existing models [305].

In a nutshell, the development of bioinformatics tools and methodologies has created new opportunities for the researchers to study tumor characteristics and its microenvironment complexities in silico. In addition to providing deeper understanding of the intrinsic heterogeneity associated with tumor development, progression and immune evasion, these tools have guided us toward therapeutic and diagnostic discoveries. By applying the current computational tools, it is now possible to predict the distinct immune and stromal components diversity within TME. Also, the available methods can create high resolution TME cell type-specific interaction network, which may eventually help in improving our understanding of cancer therapy responders and non-responders patients as well as to the development of immune-modulatory drugs.

## Conclusion

In this review, various aspects of TME, including its therapeutic potential has been described. TME heterogeneity is contributed by its highly diverse and non-uniformly distributed cellular and acellular components (Fig. 1). Variability of these components within the TME gives rise to several physiologically different specialized tumor microenvironments such as the acidic niche, hypoxic, innervated and inflammatory microenvironments. In the recent years, these specialized tumor microenvironments have emerged as the hotspots for therapeutic interventions. TME-specific therapeutic strategies can be broadly categorized based on the TME components targeted such as ECM, non-immune cells and immune cells. Major cancer immunotherapy approaches involving targeting of the immune components of TME include adoptive-T lymphocytes and CAR-based therapies, cancer vaccines, and employing immune checkpoint inhibitors. Thus, various TME components provides an opportunity to target tumor progression and metastasis through therapeutic interventions as summarized in Fig. 2. However, one of the major limitations associated to these cancer immunotherapy approaches targeting TME include primary or acquired resistance due to various extrinsic and intrinsic factors. A deeper understanding of the dynamic TME components and their real-time interaction could help in overcoming these limitations. Towards this end, several in vitro 3D experimental model systems have been developed to precisely mimic the TME conditions. Also, bioinformatics tools can be used to estimate tumor purity, immune repertoire profiling, predict neoantigens and prognostic genes in the TME. Altogether, this article gives an overview of TME components, and their promising future potential as therapeutic targets in the light of knowledge gained through experimental 3D model systems and bioinformatics predictions.

## Data Availability

Not applicable.

## Abbreviations

AML: Acute myeloid leukemia
ASCs: Adult stem cells
ATRA: All-trans retinoic acid
ALI: Air–liquid interface
APCs: Antigen-presenting cells
BTLA: B- and T-lymphocyte attenuator
BC: Breast cancer
BCRs: B-cell receptors
CAR: Chimeric antigen-receptor
CIT: Cancer immunotherapy
CTLs: Cytotoxic T lymphocytes
CAFs: Cancer-associated fibroblasts
CSSs: Cancer stem cells
CXCL12: CXC-chemokine ligand 12
CSF-1: Colony stimulating factor 1
CTLA4: Cytotoxic T-lymphocyte associated protein 4
CRS: Cytokine release syndrome
CTCs: Circulating tumor cells
CMSs: Consensus molecular subtypes
dECM: Decellularized ECM
DCs: Dendritic cells
DDR: DNA damage repair
EC: European Commission
ECM: Extracellular matrix
EMT: Epithelial-to-mesenchymal transformation
EIFs: EMT inducing factors
EndMT: Endothelial-to-mesenchymal transformation
EGF: Epidermal growth factor
FAP: Fibroblast activating protein
FGF2: Fibroblast growth factor 2
FDA: US Food and Drug Administration
GVHD: Graft-versus-host disease
GFs: Growth factors
GATK: Genome Analysis Toolkit
GSEA: Gene set enrichment analysis
HUVECs: Human umbilical vein epithelial cells
HIF-1: Hypoxia inducible factor-1
IFN-γ: Interferon-γ
IC10: Integrative cluster 10
IDH: Isocitrate dehydrogenase
irAEs: Immune-related adverse events
IDO: Indoleamine 2,3-dioxygenase
ICBs: Immune checkpoint blockers
MSCs: Mesenchymal stromal cells
MMPs: Matrix metalloproteinases
MHC: Major Histocompatibility complex
NK: Natural Killer cells
NSAID: Non-steroid anti-inflammatory drug
NSCLC: Non-small cell lung cancer
OS: Overall survival
PDGF: Platelet-derived growth factor
PDGFR: Platelet-derived growth factor receptor
PD-1: Programmed cell death protein-1
PD-L1: Programmed death ligand-1
PGE2: Prostaglandin E2
PDAC: Pancreatic ductal carcinoma
PSC: Pancreatic stellate cells
PDOs: Patient-derived organoids
PDX: Patient-derived xenografts
PSCs: Pluripotent stem cells
POLE: Polymerase epsilon
PDMS: Polydimethylsiloxane
PPI: Protein–protein interaction
pMHC: Peptide-MHC
ROS: Reactive oxygen species
TGF-β: Transforming growth factor beta
TILs: Tumor-infiltrating lymphocytes
TNF-α: Tumor necrosis factor-α
TAMs: Tumor-associated macrophages
TANs: Tumor-associated neutrophils
TLR3: Toll-like receptor 3
Tregs: Regulatory T-cells
TIGIT: T-cell immunoreceptor with Ig and ITIM domains
TCRs: T-cell receptors
TME: Tumor microenvironment
TMS: Tissue matrix scaffold
TCGA: The Cancer Genome Atlas
VEGF: Vascular endothelial growth factor
UCB: Umbilical cord blood
